# Expression of *TNFR1*, *VEGFA*, *CD147* and *MCT1* as early biomarkers of diabetes complications and the impact of aging on this profile

**DOI:** 10.1038/s41598-023-41061-0

**Published:** 2023-10-20

**Authors:** Joyce Regina Santos Raimundo, Beatriz da Costa Aguiar Alves, Jéssica Freitas Araujo Encinas, Andressa Moreira Siqueira, Katharyna Cardoso de Gois, Matheus Moreira Perez, Giuliana Petri, José Francisco Ramos dos Santos, Fernando Luiz Affonso Fonseca, Glaucia Luciano da Veiga

**Affiliations:** 1 Laboratório de Análises Clínicas do Centro Universitário–Faculdade de Medicina do ABC (FMABC), Avenida Lauro Gomes, 2000, Santo André, SP 09060-650 Brazil; 2 Departamento de Ciências Farmacêuticas da Universidade Federal de São Paulo/UNIFESP, Campus Diadema, Rua Prof. Artur Riedel, 275, Diadema, SP 09972-270 Brazil; 3 Vivarium and Animal Experimentation Laboratory-Faculdade de Medicina Do ABC (FMABC), Avenida Lauro Gomes, 2000, Santo André, SP 09060-650 Brazil

**Keywords:** Biomarkers, Nephrology, Gene expression analysis

## Abstract

Hyperglycemia leads to microvascular lesions in various tissues. In diabetic nephropathy—DN, alterations in usual markers reflect an already installed disease. The study of new biomarkers for the early detection of diabetic complications can bring new prevention perspectives. Rats were divided into diabetic adult—DMA—or elderly—DME and control sham adult—CSA—or control sham elderly—CSE. Blood and urine samples were collected for biochemical analysis. Bulbar region, cardiac, hepatic and renal tissues were collected for target gene expression studies. As result, DMA showed decreased *TNFR1*, *MCT1* and *CD147* expression in the bulbar region, *TNFR1* in the heart, *VEGFA* and *CD147* in the kidney and *TNFR1* in blood. Positive correlations were found between *TNFR1* and *MCT1* in the bulbar region and HbA1c and plasma creatinine, respectively. DME showed positive correlation in the bulbar region between *TNFR1* and glycemia, in addition to negative correlations between *CD147* in the heart versus glycemia and urea. We concluded that the initial hyperglycemic stimulus already promotes changes in the expression of genes involved in the inflammatory and metabolic pathways, and aging alters this profile. These changes prior to the onset of diseases such as DN, show that they have potential for early biomarkers studies.

## Introduction

The development of DM causes complications due to micro and macrovascular changes resulting from the formation of advanced glycation end products (AGEs) which, through binding to their receptors (RAGE), increase the generation of reactive oxygen species (ROS). ROS favor the formation of free radicals and promote oxidative stress^1^. Nephropathies, cardiovascular diseases and cerebrovascular accidents (CVA) are among the frequent complications of DM and, when associated with aging, damage tends to be more evident^2–4^. Aging and diabetes share pathophysiological molecular pathways that culminate in mitochondrial dysfunction. Therefore, metabolic changes associated with aging pave the way for the occurrence of differences between pathophysiological molecular profiles at more advanced ages^5^.

Cardiovascular complications such as diabetic cardiomyopathy, are among the leading causes of mortality in diabetic patients. Oxidative stress induces inflammatory responses and myocyte apoptosis, resulting in tissue remodeling^6^. A study in rats in which DM was induced with streptozotocin observed that treatment with insulin prevented alterations associated with diabetic cardiomyopathy, such as diastolic dysfunction and ventricular hypertrophy^7^.

Sustained hyperglycemia induces macroangiopathy and microvascular damage in brain tissue, leading to circulatory dysfunction^8^. Neuronal injury also occurs due to oxidative stress and inflammation, which lead to DNA oxidation, mitochondrial damage and activation of apoptosis^9^. A study using DM-induced rats observed high levels of lipid peroxidation and low levels of mitochondrial biogenesis in brain tissue. Furthermore, treatment with the antidiabetic syringic acid for 6 weeks improved these levels^10^.

The cellular dysfunction caused by hyperglycemia increases the risk of installed renal fibrosis, resulting in diabetic nephropathy (DN)^11,12^. DN is the main cause of chronic kidney disease (CKD) in patients referred for hemodialysis^13^; therefore, monitoring of the glomerular filtration rate (GFR) through creatinine clearance and microalbuminuria after the diagnosis of DM is recommended^14^. Likewise, urea, proteinuria and cystatin C are parameters for monitoring renal function^15–18^. However, these markers, when altered, indicate an already installed kidney disease^3^ and about 1/3 of patients experience a decline in kidney function even before the onset of proteinuria^11^.

Several studies have been carried out with biomarkers that present altered plasmatic concentrations before already established laboratory parameters for the diagnosis of DN are altered^19–22^. In an animal model, the expression of circulating vascular endothelial growth factor (VEGF), a protein involved in cell proliferation, was shown to be increased and positively correlated with DN^22,23^.

Faced with inflammatory stimuli, cells release soluble tumor necrosis factor receptors (TNFR), another inflammatory marker, which is related with stimulation of the kidney injury molecule 1 (KIM-1)^21^. Studies in humans have observed that circulating levels of tumor necrosis factor receptor type 1 (TNFR1) are good predictors of CKD in patients with type 2 DM with or without proteinuria. This marker is specific to diabetic kidney disease and does not present the same relation to other kidney diseases^24^. In an animal model, a higher expression of mRNA and TNFR1 protein was observed in the renal tissue of rats with installed DN^25^.

The cluster of differentiation 147 protein (CD147), or basigin, has been shown to be involved in renal fibrosis through the induction of metalloproteinases (MMPs) generating accumulation and remodeling of the extracellular matrix (ECM)^26^. CD147 is also involved in intracellular energy metabolism through interaction with monocarboxylate transport proteins (MCT), which mainly transport lactate^27^. Lactate derives from glycolysis and plays a fundamental role both in the production of ATP and in the signaling of cerebral angiogenesis^28^. MCT1 in the proximal tubule transports lactate to the intracellular environment in order to carry out gluconeogenesis^27^. In an animal model, negatively regulated *MCT1* expression was observed in the kidney during acidosis induced by NH4Cl^29^.

With the objective of investigating new biomarkers for the early detection of diabetes complications, such as DN, and their expression profiles during aging, we analyzed the expression levels of the *TNFR1*, *VEGFA*, *CD147* and *MCT1* markers. In addition, we sought to identify correlations between gene expressions and biochemical parameters already used in routine diagnosis and control of DN.

## Results

### Biochemical parameters

The DMA and DME groups received 120 mg/kg of the drug Alloxan, which has cytotoxicity specific to pancreatic β cells, resulting in damage to pancreatic islets and the onset of DM. In the literature, a mortality margin of up to 30% after induction with Alloxan was observed, due to its toxicity^30^. In our study, the DMA group had a mortality rate of 23% and the DME group of 25%. No difference in food intake was found between groups during the 12-h period (see supplementary data S1), however there was a significant increase in water intake during the 24-h period in the DMA versus control group (59.2 ± 33.4 n = 9 vs. 22.6 ± 7.8 n = 5 *p 0.0425) (see supplementary data S1). Data obtained from 24-h metabolic cage exposure.

Animals in the DMA and DME groups showed a decrease in mean weight gain over 5 weeks compared to their respective control groups (see supplementary data S1). Initial weight was measured on the day of induction—alloxan or sham—and followed up for 4 weeks after confirmation of DM. No difference was observed in the weight of the right kidney and heart between the diabetic groups and their controls on the day of euthanasia, but upon comparing the DMA and DME groups this difference was significant (1.03 ± 0.1 n = 9 vs. 1.3 ± 0.2 n = 11 *p 0.0049) (see supplementary data S1).

Upon confirmation of the onset of DM, the diabetic rats had higher glycemic and HbA1c values compared to controls. Regarding the data evaluating renal function, all groups presented values within the reference range, with the exception of urea, which indicates that in the present model there was no installation of DN. However, plasma urea and creatinine concentrations were statistically higher in the DMA and DME groups, and urinary creatinine showed lower values compared to their respective control groups. Creatinine clearance showed significantly lower values only in the DME group compared to controls (Table 1).

**Table 1:** Laboratory parameters.

Parameters	CSA	DMA	*p	CSE	DME	*p
Glycemia (mg/dl)	132.4 ± 45 n = 5	462.7 ± 98.0 n = n = 9	0.001*	106.8 ± 9.2 n = 9	447.4 ± 125.7 n = 7	0.0002*
HbA1c (%)	4.5 ± 0.2 n = 5	9.0 ± 2.0 n = 8	0.0016*	4.6 ± 0.05 n = 5	9.3 ± 1.3 n = 4	0.008*
Ureia (mg/dL)	48.5 ± 6.9 n = 5	135.5 ± 38.3 n = 6	0.0043*	45.4 ± 9.1 n = 9	93.2 ± 53.8 n = 7	0.023*
Plasma crea. (mg/dL)	0.6 ± 0.1 n = 5	0.7 ± 0.1 n = 6	0.0065*	0.5 ± 0.1 n = 9	0.7 ± 0.1 n = 7	0.01*
Urine crea. (mg/dL)	60.7 ± 11.2 n = 5	21.7 ± 10.3 n = 9	0.001*	63.2 ± 40.8 n = 9	26.4 ± 14.5 n = 6	0.0360*
Creatinine clearance	0.3 ± 0.2 n = 5	0.2 ± 0 n = 4	0.1	0.6 ± 0.2 n = 5	0.3 ± 0.1 n = 4	0.0317*
Proteinuria (mg/dl)	16 ± 3.1 n = 5	12.5 ± 2.3 n = 9	0.0460*	28.3 ± 15.7 n = 9	19.5 ± 11.4 n = 6	0.11
Microalbum (mg/24)^a^	0.2 ± 0.4 n = 4	0.9 ± 1.8 n = 9	> 0.9999	2 ± 4.5 n = 9	10.3 ± 20.4 n = 6	0.95
Apo B/A ratio	1.2 ± 0.7 n = 5	0.8 ± 0.2 n = 6	0.6623	0.8 ± 0.3 n = 9	0.4 ± 0.2 n = 6	0.1
Cystatin C (mg/L)	0.2 ± 0.01 n = 5	0.2 ± 0.1 n = 4	> 0.9999	0.17 ± 0.01 n = 6	0.2 ± 0.1 n = 4	0.14

^a^Most microalbuminuria values were zero. Values presented as mean ± DSVP and number of samples. Mann–Whitney and Student’s *t*-test *P < 0.05. 95% CI. Reference values for Wistar rats: Glycemia: 72–193 mg/dL; HbA1c: 4–6%; Urea: 45–80 mg/dL; Plasma Crea.: 0.24–1.20 mg/dL; Urine Crea.: 32–97.4 mg/dL; Creatinine clearance: 0.2–1.8 mL/min; Proteinuria: 8.8–33.2 mg/dL; Microalbuminuria: < 20 mg/24; Apo B/A Ratio: 0.7–1.2; Cystatin C: 0.18–1.5 mg/dL^30–35^.

### Expression profile of the studied genes

**Figure 1:**
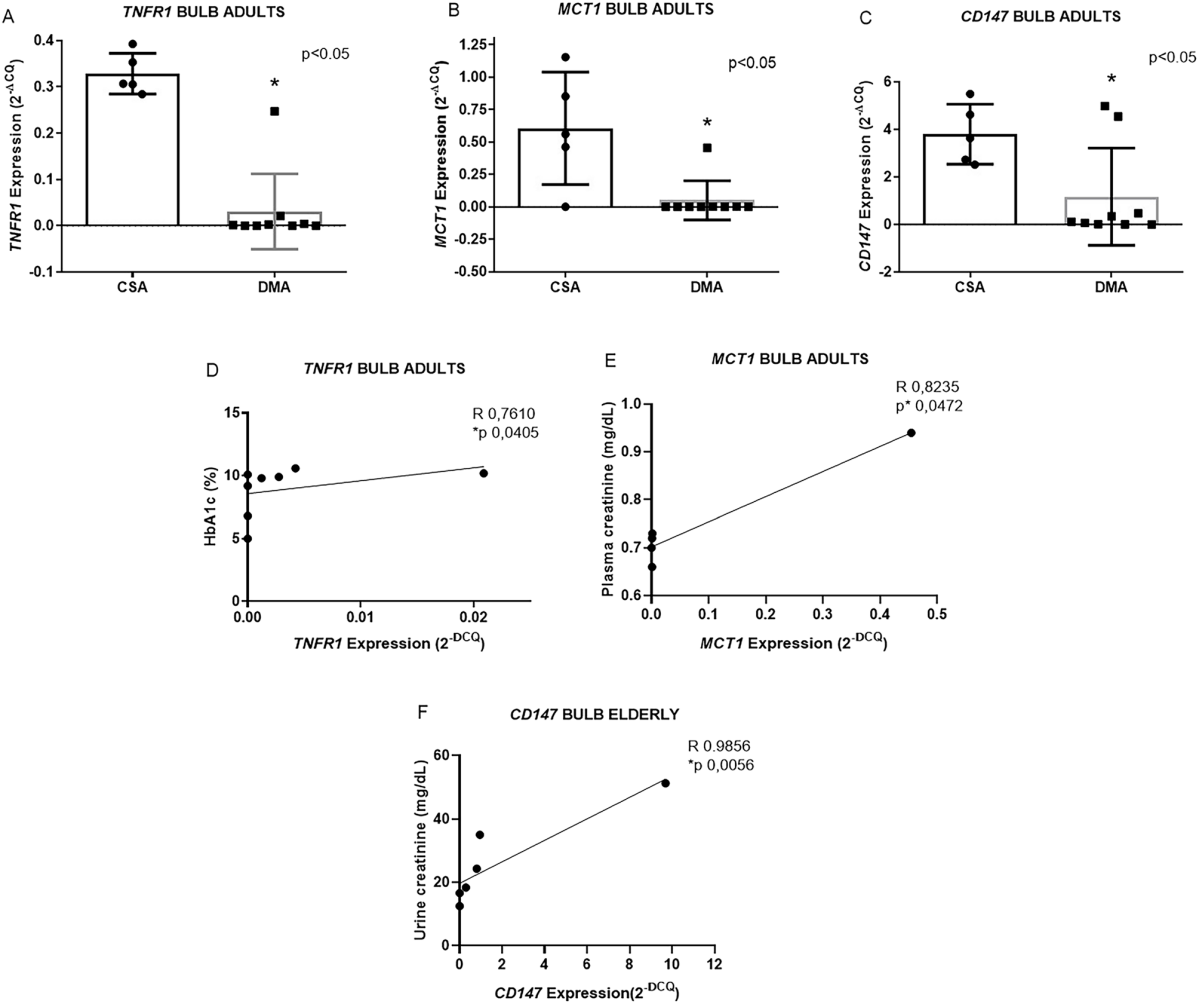
Graphs representing *TNFR1* (**A**), *MCT1* (**B**) and *CD147* (**C**) gene expression in the bulbar region and positive correlations of bulbar region *TNFR1* (**D**), *MCT1* (**E**) expression in the DMA group. Representative graph of the positive correlations of *CD147* expression in the heart of the DME group (**F**). Data expressed as mean ± DSVP. Mann–Whitney and Spearman correlation tests. *p < 0.05 vs. control. 95%CI.

In the bulbar region of the DMA group, no alteration was observed in *VEGFA* expression (data not shown—see Additional file 3), however there was decreased expression of *TNFR1* (0.1 ± 0.2 n = 5 vs. 0.03 ± 0.08 n = 9 *p 0.001), *MCT1* (0.6 ± 0.4 n = 5 vs. 0.05 ± 0.1 n = 9 *p 0.0380) and *CD147* (3.8 ± 1.2 n = 5 vs. 1.1 ± 2.04 n = 9 *p 0.0420) (Fig. 1A–C). The DME group showed no statistical differences between the studied genes (see supplementary data S2). In addition, positive correlations were observed in the DMA group between *TNFR1* in the bulbar region and HbA1c (R 0.7610 and *p 0.0405 n = 8), *MCT1* in the bulbar region and plasma creatinine (R 0.8235 and p* 0.0472 n = 6) and between CD147 and urine creatinine (Fig. 1D–F). In the DME group, we observed a positive correlation for *CD147* in the bulbar region and urine creatinine (R 0.9856, *p 0.0056 n = 6) (Fig. 1F). It is possible to observe the global expression means of *TNFR1* in the studied tissues (see supplementary data S2).

**Figure 2:**
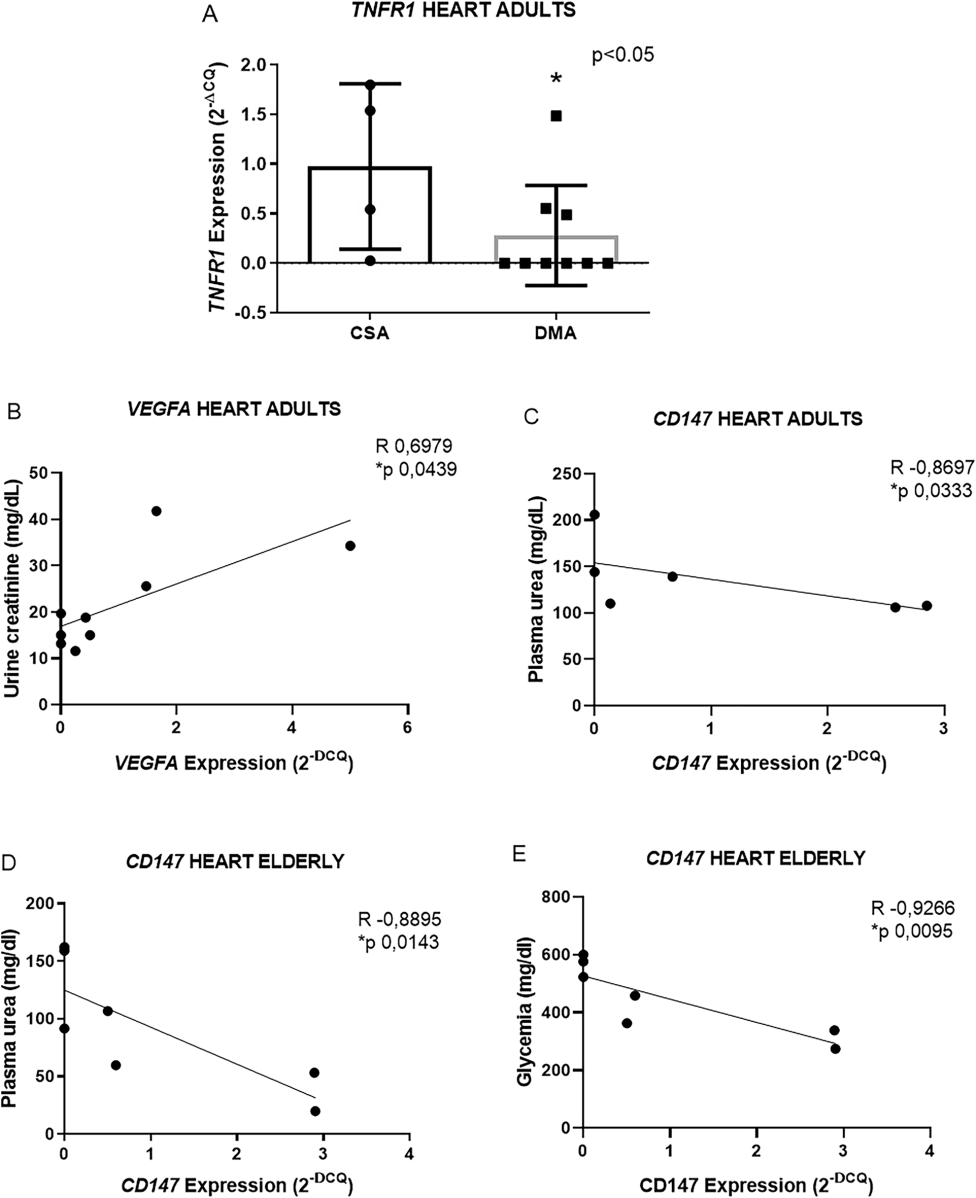
Representative graphs of *TNFR1* gene expression in the heart of the adult group (**A**). Graph representing the positive correlation between *VEGFA* in the heart and urine creatinine (**B**), and the negative correlation between *CD147* in the heart and plasma urea (**C**), in the DMA group. Representative graph of the negative correlations between *CD147* in the heart with plasma urea and glycemia in the DME group (**D**,**E**). Data expressed as mean ± DSVP. Mann–Whitney and Spearman correlation tests. *p < 0.05 vs. control. 95% CI.

In the heart, *TNFR1* expression was lower in the DMA group compared to the CSA group (0.9 ± 0.8 n = 5 vs. 0.3 ± 0.5 n = 9 *p 0.0420) (Fig. 2A), while no changes in expression were observed for the other genes (see supplementary data S2). The DME group did not show altered expression of the evaluated genes (see supplementary data S2). In the CSA and CSE groups, obtaining *MCT1* gene expression values was not possible, thus precluding the comparative statistical analysis. Furthermore, in the DMA group we observed a positive correlation between *VEGFA* expression in the heart and urine creatinine (R 0.6979 and *p 0.0439 n = 9;) and a negative correlation between *CD147* in the heart and urea (R -0, 8697 and *p 0.0333 n = 6) (Fig. 2B and C). In the DME group, we observed negative correlations between *CD147* in the heart versus urea (R -0.8895 and *p 0.0143 n = 7) and glycemia (R -0.9266 and *p 0.0095 n = 7) (Fig. 2D and E).

**Figure 3:**
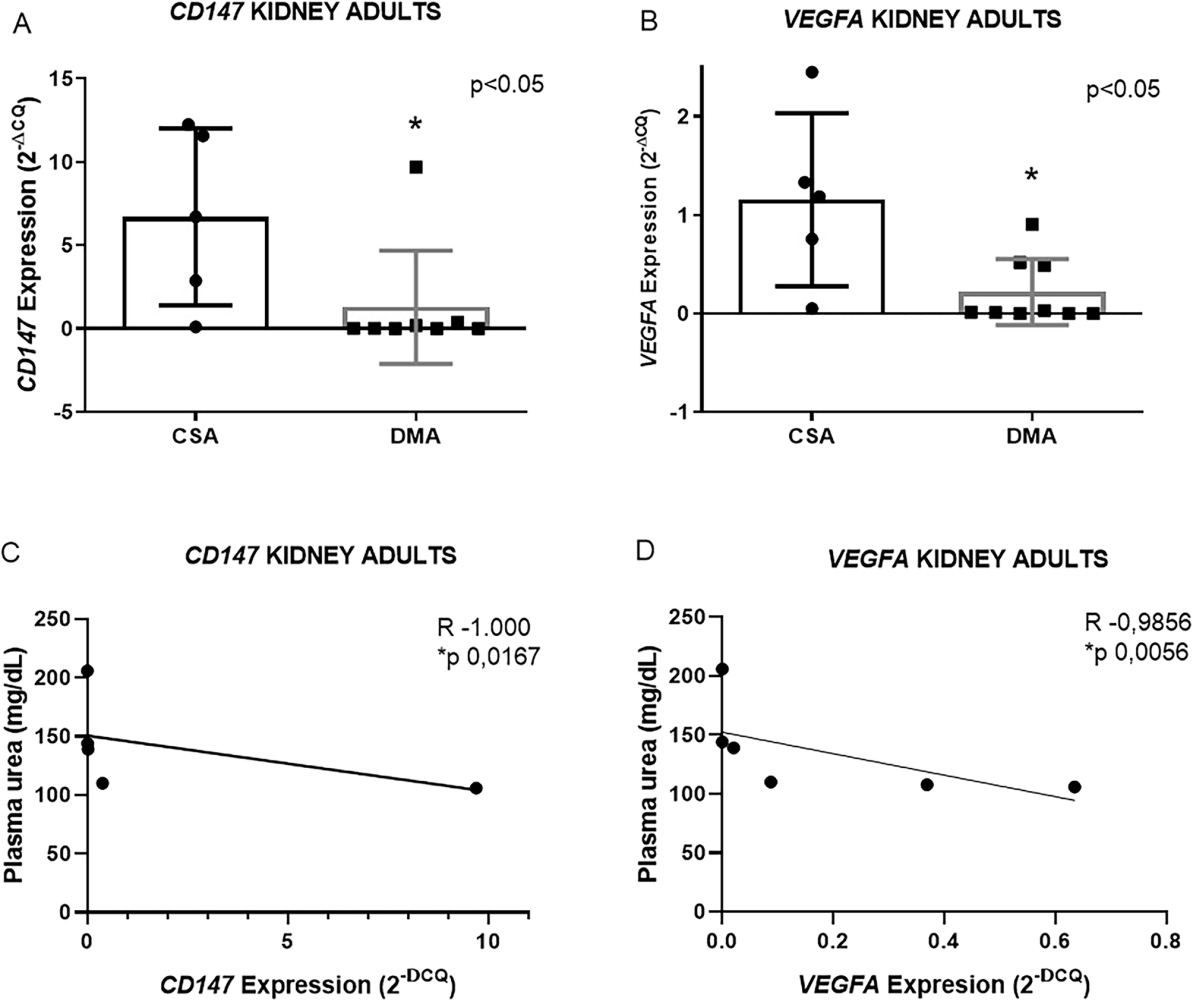
Representative graphs of *CD147* and *VEGFA* expression in the kidney of the DMA group (**A**,**B**) Representative graphs of the negative correlations between *CD147* and *VEGFA* in the kidney with plasma urea and glycemia in the DMA group (**C**,**D**). Data expressed as mean ± DSVP. Mann–Whitney and Spearman correlation tests. *p < 0.05 vs. control. 95% CI. Spearman correlation test. *p < 0.05.

In kidneys of the DMA group, decreased *CD147* (6.7 ± 5.3 n = 5 vs. 1.3 ± 3.4 n = 9 *p 0.0287) and *VEGFA* (1.1 ± 0.8 n = 5 vs 0.2 ± 0.3 n = 9 *p 0.0120) expression was observed (Fig. 3A and B) while the other genes showed no differences when compared to controls (see supplementary data S2). In the DME group, it was only possible to evaluate the expression of *CD147* in the kidney (see supplementary data S2) and the *TNFR1*, *VEGFA* and *MCT1* genes were not expressed in some tissues of the CSE group, thus precluding the comparative statistical analysis in the elderly group. Furthermore, the DMA group showed a negative correlation between *CD147* in the kidney and urea (R − 1000 and *p 0.0167 n = 5) and *VEGFA* in kidney tissue and urea (R − 0.9856 and *p 0.0056 n = 6) (Fig. 3C and D).

**Figure 4:**
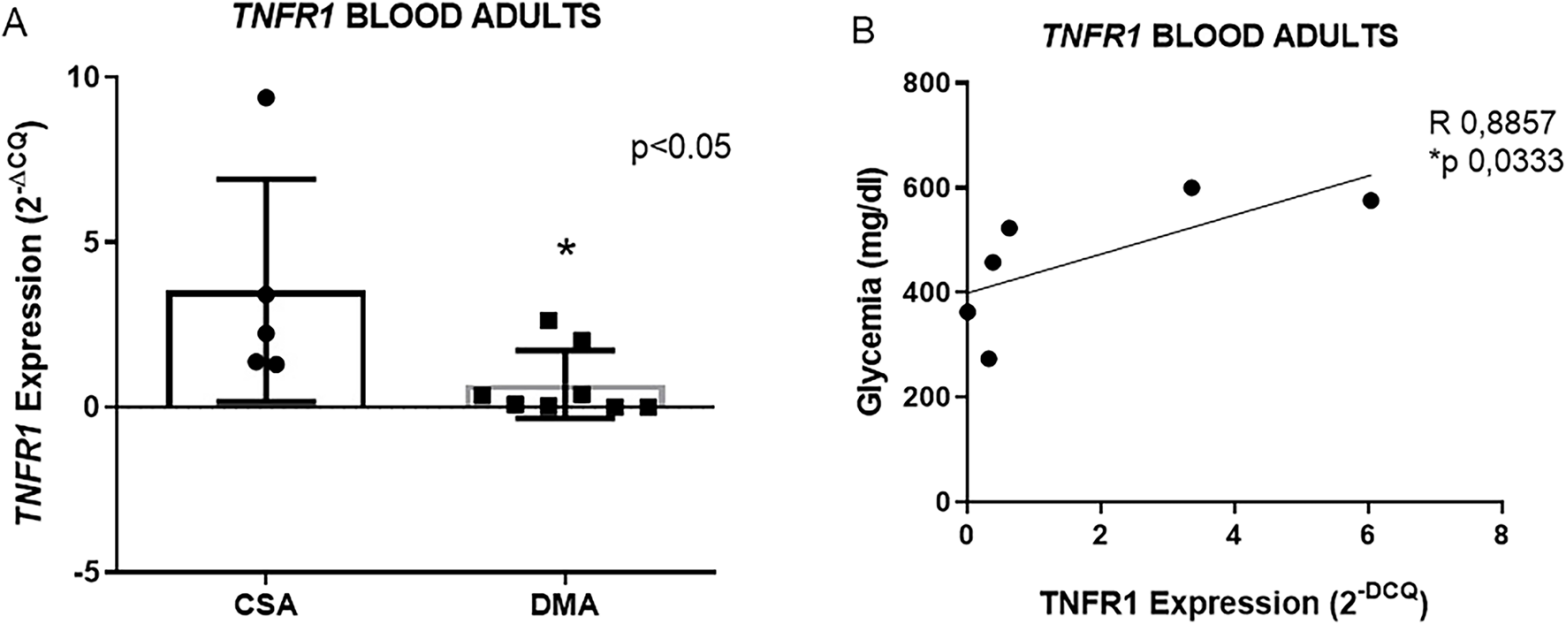
*TNFR1* in the peripheral blood collected from the adult group (**A**). Graph representing the positive correlation between *TNFR1* in the blood and glycemia in the DME group (**B**). Data expressed as mean ± DSVP. Mann–Whitney and Spearman correlation tests. *p < 0.05 vs. control. 95% CI.

In blood samples, *TNFR1* expression was lower in the DMA group compared to the CSA group (3.5 ± 3.3 n = 5 vs. 0.7 ± 1.0 n = 9 *p 0.0295) (Fig. 4A) and no changes were observed in the expression of the other genes (see supplementary data S2). The DME group did not show alterations in the expression of the studied genes compared to the control (see supplementary data S2). We neither observed *VEGFA* expression in some samples of the CSA and CSE groups, nor *MCT1* in some samples of the CSE group, therefore precluding the comparative statistical analysis. Finally, in the DMA group, we observed a positive correlation between *TNFR1* in the blood and glycemia (R 0.8857 and *p 0.0333 n = 6) (Fig. 4B).

In the liver, no alterations in the expression of the studied genes were observed in the DMA and DME groups compared to their respective controls (see supplementary data S2). Additional file 4 shows the gene expression profile in all the studied groups and tissues for *TNFR1*, *VEGFA*, *CD147* e *MCT1* (see supplementary data S3).

## Discussion

The objective of the present study was to investigate the gene expression profile of new candidates for early biomarkers for DN, as well as how they correlate with the commonly used parameters for assessing renal function. Biochemical evaluations did not determine the installation of DN. The obtained plasma urea and urine creatinine values showed oscillations that may be associated with variations in environmental, stress, genetic and methodological factors^34^. Despite the biochemical dosages presenting values within the reference range, with the exception of urea, the diabetic groups showed differences in some parameters when compared with their respective controls. Increased urea reflects the increase in its reabsorption due to the greater volume of urine^36^ and lower urinary excretion and the consequent higher concentration of plasma creatinine, leading to a reduction in its clearance^37,38^.

**Figure 5:**
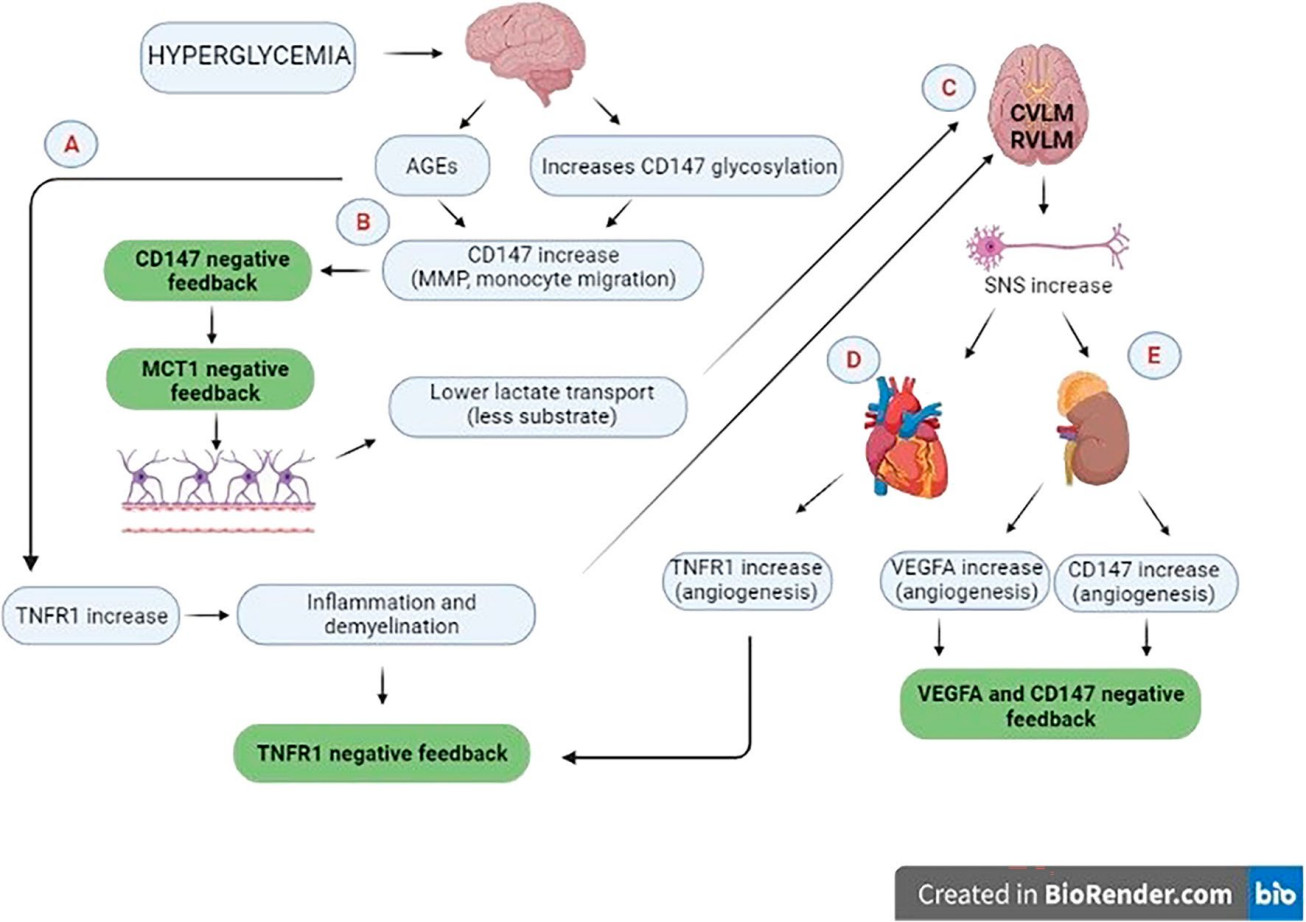
The figure shows the gene expression alterations found and generation of hypotheses. The increased formation of AGEs in the bulbar region leads to greater stimulation of *TNFR1*, which in response undergoes negative regulation (**A**). Likewise, *CD147* is increased, which, when undergoing negative feedback, also negatively regulates *MCT1* expression (**B**). The damage caused by the increase in *TNFR1* and the reduction in lactate transport in the bulbar region may compromise the efferent pathways of the SNS (**C**), whose increase could stimulate *VEGFA* in the kidney and *CD147* in the renal and cardiac tissue, again triggering negative feedback at this early-stage DM (**D** and **E**). Created by author in the BioRender website.

In the molecular analyses, a reduction in *TNFR1*, *CD147* and *MCT1* expression was observed in the bulbar region in the DMA group. In DM, the greater production of AGEs is known to be directly related to the formation of ROS and the installation of persistent inflammatory processes, in which the activation of the TNF system plays an important role^39,40^. Increased *TNFR* activation is directly involved in the demyelination process, for example^41^. In the early stages of inflammation and with the expression of some genes, noxious cellular stimuli can trigger inhibitory mechanisms^42,43^. Perhaps the decrease in *TNFR1* expression reduces its availability in cell membranes in order to attenuate harmful activation of the TNF system, functioning as a negative feedback system (Fig. 5).

AGEs also stimulate the CD147 protein, which promotes increased expression of MMPs, involved in the degradation of ECM and in the alteration of its tissue deposition, leading to the remodeling and stimulation of monocyte migration^44^. Therefore, a decrease in *CD147* expression would compensate for this damage during this initial phase of DM onset. Furthermore, it has already been demonstrated in an in vitro model of rheumatoid arthritis that there is co-regulation between *TNFR1* and *CD147*. The authors describe that the negative regulation of *CD147* decreases the secretion of cytokines derived from the NF-κB pathway, which in turn is activated by the binding of TNF to its TNFR1 receptor^45^.

CD147 also plays an important role in the neuronal metabolic pathway. In astrocytes, the production of lactate is derived from glucose metabolism, which is stored and delivered to neurons as a metabolic source. The lactate transport proteins MCT1 and MCT4 are present in astrocytes while MCT2, in neurons. Since CD147 acts as a chaperone for MCT1 and 4, the decrease in *MCT1* expression and its chaperone may indicate lower lactate transport and lower energy for neurons^46^. Monocarboxylate disorders have already been associated with pathologies such as neurodegeneration, cognitive alterations, epilepsy and metabolic disorders^28^. In this case, another hypothesis proposed in this work is that the lower availability of neuronal metabolic fuel in the bulbar region—the main control nucleus of the cardiovascular system—could be the primary mechanism responsible for disturbances in the firing of pre-motor neurons in the sympathetic nervous system (Fig. 5). However, more specific studies need to be carried out to prove this hypothesis.

In the heart, *TNFR1* expression was also reduced in the DMA group. Activation of TNFR1 in myocytes in vitro is known to exacerbate pathological remodeling in heart failure after myocardial infarction^47^. TNFR1 also plays an important role in the complications of DM and the development of cardiovascular diseases, and is related to greater induction of apoptosis^48^.

In the kidney, *VEGFA* gene expression showed decreased values in the DMA group. VEGFA is known to be a pro-angiogenic factor that increases vascular permeability and contributes to the endothelial dysfunction that occurs in DM. In rats with DN, increased *VEGFA* expression has been shown to be related to increased cell proliferation and permeability of endothelial cells, which contribute to accumulation in the ECM^49^. An increase in *VEGFA* expression in podocytes was observed in rats 35 days after undergoing DM induction with streptozotocin^50^. However, these animals already had proteinuria, while in the present study no change in this parameter was observed. This result can be attributed to methodological differences related to the drug used in DM induction and tissue collection time. On the other hand, Veron et al. demonstrated that diabetic *VEGFA* knockdown mice develop diffuse gomerulosclerosis and increased glomerular basement membrane thickness^51^.

In the kidney, *CD147* was reduced in the DMA group. Since this protein is involved in the increase of MMPs and accumulation of ECM, its increased concentration can lead to the disruption of the integrity of podocytes and the glomerular basement membrane. Furthermore, *CD147* upregulates *VEGF* production in adriamycin-induced nephropathy, indicating a co-regulatory mechanism^52^. In humans, plasma CD147 concentration has been directly associated with DN progression^53^. In renal carcinoma, higher *CD147* expression has been shown to be related to higher carcinogenesis and increased lactate transport to the intratumoral environment^54–56^. Among the data that were raised in the literature, this is the first work to investigate *CD147* gene expression in the context of diabetic nephropathy in rats.

In blood samples, *TNFR1* gene expression was reduced in the DMA group. The concentration of circulating TNFRs has been shown to be related to lower GFR values, thus with potential for predicting decline in renal function^57^. TNFR1 is a soluble receptor that has shown, in obese diabetic mice, to have its plasmatic concentration increased along with TNF-α levels. In addition, in vitro TNF-α stimulation has been shown to increase TNFR expression in renal tubular cells^58^, therefore suggesting that in the early stages of exposure to hyperglycemia, the expression of *TNFR1* mRNA is already altered, evidencing its predictive capacity for kidney damage. In fact, altered expression of this marker in adult rats presented a homogeneous profile both in the blood and in the bulbar region and heart, shaping a scenario demonstrating the potential of TNFR1 as a marker in these tissues^59^.

It is interesting to note that the specificity of TNFRs in predicting the progression of DN is not observed in other renal pathologies, making it a strong candidate for a predictive biomarker^24^. The mechanisms by which TNFRs initiate and sustain kidney injury have not yet been fully elucidated, however the present study provides evidence that this gene already shows changes in its expression in the early stages of DM, when DN has not yet been established. We believe that the studied model represents a period of DM where negative feedback of *TNFR1* expression occurs in some tissues, with the aim of preserving cells against the persistent inflammatory stimulus characteristic of hyperglycemia^57^.

The present study encountered limitations in the expression analysis of certain genes. TNFR1 and VEGFA were found to be unexpressed in the kidney of the CSE group, and VEGFA was not detected in blood samples from both the CSA and CSE groups. Additionally, obtaining MCT1 expression values in heart tissues of the CSA and CSE groups, as well as in the kidney and blood of the CSE group, was not possible. These limitations hindered comparative statistical analysis and restricted the assessment of a broader range of gene expression. These challenges may arise from low concentrations of genetic material in the samples and inherent sensitivity limitations of the employed techniques^60–62^.

In the diabetic groups, analysis of the correlation between expression of the studied genes and biochemical parameters suggests that expression of *TNFR1*, *CD147*, *MCT1* and *VEGF* is modulated by glycemia, creatinine and urea, in the bulbar region, cardiac and renal tissues. This evidences that metabolic disturbances present in the onset of DM are important in the molecular alterations associated with the pathophysiology of DN. In this study, *TNFR1* demonstrated a positive correlation with glycemic values, and studies have already shown that this marker is positively modulated in DM^63,64^. However, negative regulation of its expression may occur in the earlier stages of the disease, as previously discussed.

Creatinine and urea seem to stimulate negative modulation of *CD147* and *VEGF* expression, thus reducing the pathological induction of MMPs and pro-angiogenesis^49^. In the case of *MCT1*, the positive correlation with plasma creatinine may indicate that it stimulates increased lactate transport in the brain. In a human model of severe non-cerebral malaria, increased creatinine was associated with greater susceptibility to cytotoxic edema^65^, where there is greater release of lactate for neuronal nutrition^66^. However, in this studied model, *MCT1* showed reduced expression in the bulbar region of the DMA group, which may be related to the decreased expression of its chaperone *CD147*. Therefore, the study of the expression of these genes, which are related to the intracellular inflammatory and metabolic pathways, represents potential in the search for candidates for early markers of DN prediction and prognosis^63,64^.

The pattern of reduced *TNFR1*, *MCT1*, *CD147* and *VEGFA* gene expression that was observed in the DMA group was not seen in the DME group. This result is related to the absence of expression of these genes in some tissues from the elderly group, however the endogenous gene was expressed, indicating tissue viability. Some studies associate alterations in the expression of Micro RNAs (miRNA) with the silencing of multiple genes during aging.

Several miRNAs, such as miR-29, miR-181a, and miR-222, have been shown to be differentially regulated during aging^67,68^. Likewise, it has been observed in DN that several miRNAs, such as MiR-122-5p, Mir-98 and MiR-136, regulate the expression of several genes involved in renal fibrosis^69–71^. The expression of miRNAs should be studied in future stages of this line of research to confirm this hypothesis.

In the initial stages of chronic diseases, physiological mechanisms of negative or positive regulation are observed, in an attempt to compensate for the metabolic imbalance. A classic example of this compensation mechanism was described by Guyton and Hall in the early stages of hypertension. In this case, renal filtration is increased in order to reduce blood pressure. However, consequently there is damage to the glomerular capillaries and gradual loss of renal function^72^. In the present study, the biochemical and expression profile of some genes involved in the pathophysiology of DN can be outlined. A general pattern of reduction in these expression profiles was observed, therefore, based on the theory of Guyton and Hall, we believe that in the initial stages of DM this negative regulation occurs in metabolic and inflammatory pathways in order to reduce the harmful stimuli of hyperglycemia and preserve the tissue (Fig. 5).

## Conclusion

The present study demonstrated that the noxious hyperglycemic stimulus in the initial phase of DM promotes a reduction in *TNFR1* and *CD147* expression in several tissues, in addition to a decrease in *MCT1* expression in the bulbar region and *VEGF* in the kidney, revealing a negative feedback profile. Furthermore, the expression of these genes has been shown to correlate with biochemical parameters used for diagnosing and monitoring DN. The important role of the proteins encoded by these genes in this pathophysiology and other complications of diabetes is known. Thus, altered expression in these genes, prior to signs of installation of these secondary diseases, show that these biomarkers have the potential to reveal changes at an early stage, with special attention to TNFR1, which has specificity for DN. Possibly, the first alterations in gene regulation occur, more evidently, in adult animals. Therefore, this study also suggests that during aging, the proposed negative feedback may be altered due to molecular changes associated with advanced age.

## Methods

### Arrive guideline statement

This research project was carried out after being approved by the Ethics Committee on Animal Use of ABC Health University Center—ABC Medical School, under number 20/2018.

All methods were performed in accordance with the Manual of Good Laboratory Practices. Manipulation of *Wistar* rats was carried out in accordance with the Guidelines of the Committee on Ethics in the Use of Animals. This study is reported in accordance with ARRIVE guidelines.

### Animals and experimental model

Male rats of the species *Rattus norvegicous* of the Wistar strain were placed in the housing facility of University Center FMABC in the following conditions: (a) light/dark cycle of 12 h; (b) room temperature at 21 ± 2 °C; (c) ad libitum supply of water and rodent chow; animals were left to habituate for at least 5 days prior to carrying out the experiments. At 2 months of age this species enters the reproductive phase and is considered a young adult. At 6 months there is a decline in this phase, characterizing more advanced age. Thus, rats were divided into the following experimental groups: DMA: adult animals with approximately 3 months of age, evaluated 30 days after DM induction with Alloxan. DME: elderly animals with approximately 7 months of age, evaluated 30 days after DM induction with Alloxan. CSA: adult animals with approximately 3 months of age, evaluated 30 days after saline solution injection. CSE: elderly animals with approximately 7 months of age, evaluated 30 days after saline solution injection.

### DM induction and glycemic monitoring

At the end of the habituation period, alloxan at the dose of 120 mg/Kg was administered via intraperitoneal injection^73,74^. Rats that presented glycemia above 250 mg/dL after 7 days of DM induction were selected. The animals were followed up for 30 days without treatment, where their weight and glycemic values were measured using a commercial glucometer (Accu-chek Advantage, Roche Diagnostics, Indiana, USA) every 7 days in blood collected via puncture of the caudal vein. The cytotoxicity of Alloxan is specific to pancreatic β-cells, causing damage to pancreatic islets and blood vessels, in addition to cell death. Alloxan is a drug with cytotoxicity affecting β cells and induces DM according to dose, infusion rate, route of administration, diets, fasting time and animal weight^75,76^.

### Metabolic cage

After a monitoring period of 30 days, the animals were placed in individual metabolic cages for a period of 24 h to collect urine for biochemical analyses. Urine collection was performed using recipients containing RNA later, which were positioned in the conical outlet located below the cage. Animals underwent a fasting period of 12 h prior to collection to minimize urine contamination by the feed. Water intake (mL) and urine excretion (mL) were quantified. Before collection at 24 h, animals underwent 2 days of habituation in which they remained 12 h in the metabolic cages.

### Sample collection

After urine collection at 24 h, rats were euthanized with thiopental. Immediately after administration, with the animal still alive, blood samples were collected in a tube containing EDTA for the study of gene expression and HbA1c dosage, and in a dry tube for serum separation and determination of biochemical parameters. The left kidney was extracted and fixed in formalin for later histological and histochemical analyses. In addition, the right kidney, liver, heart and brainstem bulbar region were collected to study the expression of the genes of interest.

### Determination of biochemical and renal function evaluations

#### Glycemia and HbA1c

Blood glucose was determined weekly using the photometric method and confirmed using fluoridated plasma by the automated enzymatic-colorimetric method, BioSystems Glucose Ref. COD 12503. HbA1c was determined using the immunoturbidimetry method, BioSystems Hemoglobin A1C-Turbi kit Ref. COD 13044. Dosages were performed using a COBAS 8.000 apparatus.

#### Plasma urea

Urea was determined by the enzymatic/colorimetric method using the Urea UV SL Elitech kit Cat. nº URSL-0500 (Elitech Group Clinical Systems, France) according to the manufacturer's protocol, in a COBAS 8,000 device at an absorbance of 600 nm.

#### Serum and urine creatinine

Biochemical analyses of serum and urine creatinine were determined using the kinetic-colorimetric Jaffé alkaline picric method, Creatinine Jaffe Elitech Cat. nº CRCO-0600 (Elitech Group Clinical Systems, France), according to the manufacturer's protocol, in a COBAS 8,000 device at an absorbance of 510 nm. Urine was previously homogenized and centrifuged at 2500 rpm for 5 min at room temperature to precipitate the sediment. The supernatant was diluted (1:25) in distilled water using glass tubes.

#### Creatinine clearance

Creatinine clearance was calculated using the formula Ccr = (Ucr × Vu/Pcr) × Ma. Where: Ucr = urine creatinine concentration (mg/dl); Uv = urine volume (ml/min); Pcr = plasma creatinine concentration (mg/dl); Ma = animal weight (kg)^77^.

#### Proteinuria

Determination of urinary protein excretion was performed through the reaction with copper (II) ions in an alkaline medium, resulting in a colored complex whose intensity was quantified in a COBAS 8,000 device at an absorbance of 545 nm. The kit used was Protein (Total) BioSystems (BioSystems S.A., Spain) Cat. No. COD 12500.

#### Microalbuminuria

Determination of microalbuminuria in the urine collected at 24 h was performed using the immunoturbidimetry method, with the Albumin (Microalbuminuria) BioSystems kit (BioSystems S.A., Spain) Cat. No. COD 13324, according to the manufacturer's protocol, in a COBAS 8.000 apparatus.

#### ApoA, ApoB in plasma and urine

Determination of apo B and apo A-I were performed using the turbidimetry method, with BioSystems reagents Apolipoprotein B Turbidimetry Ref. COD 31099 and BioSystems Apolipoprotein A-I Turbidimetry Ref. COD 31096, respectively. All dosages were determined using a COBAS 8000 device, using control sera to verify assay performance.

#### Cystatin C

Cystatin C was quantified using the Enzyme Linked Immunosorbent Assay (ELISA) method, Kit Cystatin C, catalog ALX-850-292, brand Enzo Life Sciences. The test is based on the identification of antigens by antibodies marked with an enzyme, which acts on its substrate and causes a change in the color of the chromogen (a colorless substance that changes color when oxidized by the enzyme).

### Tissue preparation for molecular biology analysis

Blood Lysis Buffer 1x (1550 mM NH4Cl, 100 mM KHCO3, 10 nM EDTA pH 7.4) was added to EDTA tubes containing blood sample at a ratio of 3:1 to remove red blood cells. Each tube was then homogenized for 30 min under refrigeration and centrifuged twice at 2500 rpm for 15 min at 4 °C, followed by discarding of the supernatant. The leukocyte pellet obtained after centrifugation was dissolved in 1000 μL of TRIzol Reagent Invitrogen Cat. nº 15596026 (Invitrogen Thermo Fisher Scientific—USA) and the protocol for RNA extraction was followed. Right kidney, heart and bulbar region tissues were collected in cryovials containing 1% PBS and immediately frozen at – 80 °C. Subsequently, the tissues were macerated in buffer solution with TissueRuptor II Cat. No./ID: 990890 equipment (Qiagen, Germany) and 300 μL were added to 1000 μL of TRIzol and the protocol for RNA extraction was followed. Total RNA concentration and the 260/280 ratio were estimated through spectrophotometric reading in the NanoDrop Lite equipment (GE Health Care).

### Synthesis of DNA complementary to messenger RNA (cDNA)

RNA samples were diluted to a concentration of 1 μg and cDNA synthesis was performed with the QuantiTect Reverse Transcription kit Cat No./ID: 205313 (Qiagen, Germany), according to the manufacturer's protocol.

### q-PCR for evaluation of gene expression

Expression of the *TNFR1*, *VEGFA*, *CD147* and *MCT1* genes was evaluated by real-time PCR (qPCR). To normalize the expression values of the target genes, glyceraldehyde-3-phosphate dehydrogenase (*GAPDH*) expression was used as a reference gene. The specific primers for each selected gene were designed using Primer3 Input 0.4.0 software, available at http://frodo.wi.mit.edu/primer3/. The designed primer sequences were checked for specificity using the Primer-BLAST program, available at http://www.ncbi.nlm.nih.gov/tools/primer-blast (Table 2).

**Table 2:** Sequences of specific primers and their amplicons.

Gene	Sequence	Amplicon (bp)
*GAPDH*	F-ccatcaccatcttccaggag	102
	R-tctccatggtggtgaagaca	
*TNFR1*	F-ctgtgccgatatccccagtg	115
	R-aggctggagttaggggctta	
*VEGFA*	F-ccacccacatacacacacata	130
	R-gcctctctaagaaggacgaaag	
*MCT1*	F-gctgggaagtctgatgcaa	162
	R-agcccaaagaacatgaccaca	
*CD147*	F-aagaatgtgcgccagaggaa	169
	R-gctctgtcttcacttgggct	

The real-time amplification reactions were performed with an Applied Biosystems 7500 Real Time PCR Systems thermal cycler (Applied Biosystems, United States), in a final reaction volume of 15 μL and containing: 1× SYBR Green mix (Quantitec SYBR Green PCR Cat kit No./ID: 204343, Qiagen, Germany), 1.5 μL of cDNA and 10 pmol of each specific primer in the following concentrations: *GAPDH*, *VEGFA* and *MCT1* at 0.2 µMol; *TNFR1* and *CD147* at 0.15 μMol. The thermal profile was determined with an initial heating step at 95 °C for 10 min, followed by 45 repetitions at 95 °C for 15 s and at 60 °C for 25 s. Genes that did not yet have standardized concentrations were previously determined and amplicon sizes were confirmed by 2% agarose gel electrophoresis (see supplementary data S4).

Calibration curves for each gene were performed with serial dilutions (1, 1:10 and 1:100) of cDNA synthesized from a concentration of 1 μg of total RNA obtained from control leukocytes. Gene expression was calculated by applying the 2 ^(−ΔCq)^ formula^78,79^.

### Statistical analysis

The results of gene expression and biochemical values were expressed as mean ± standard deviation (DSVP). Student’s *t*-test was performed for the CSA versus (vs.) DMA group and between the CSE vs DME group for parametric data and the Mann–Whitney test for non-parametric data. Spearman's correlation analysis was carried out in the DMA and DME groups, between the expression of the researched genes and the performed biochemical dosages. For multivariate comparison analysis between all the studied groups, the Kruskal–Wallis one-way analysis of variance test was used. All analyses were performed using the GraphPad Prism computational program (GraphPad, version 7.0, USA). The established significance level was 5% (descriptive value of p < 0.05), 95% confidence interval (CI).

### Ethics approval

This research project was carried out after being approved by the Ethics Committee on Animal Use (CEUA) of ABC Health University Center–ABC Medical School, under number 20/2018.

### Supplementary Information

Supplementary Information 1.

Supplementary Information 2.

Supplementary Information 3.

Supplementary Information 4.

## Data Availability

The datasets generated and/or analyzed during the current study are available in the ArrayExpress repository under the number E-MTAB-13000, link: https://www.ebi.ac.uk/biostudies/arrayexpress/studies/E-MTAB-13000.
