# Supplementary material for: Expression of TNFR1, VEGFA, CD147 and MCT1 as early biomarkers of diabetes complications and the impact of aging on this profile
Source: Sci Rep. 2023 Oct 20;13:17927. doi: 10.1038/s41598-023-41061-0 (PMC10589356; doi:10.1038/s41598-023-41061-0)
Supplement: Supplementary file 1 — Supplementary Information 1. [file 41598_2023_41061_MOESM1_ESM.pdf]

Food and water consumption, weight gain and heard and kidney weight

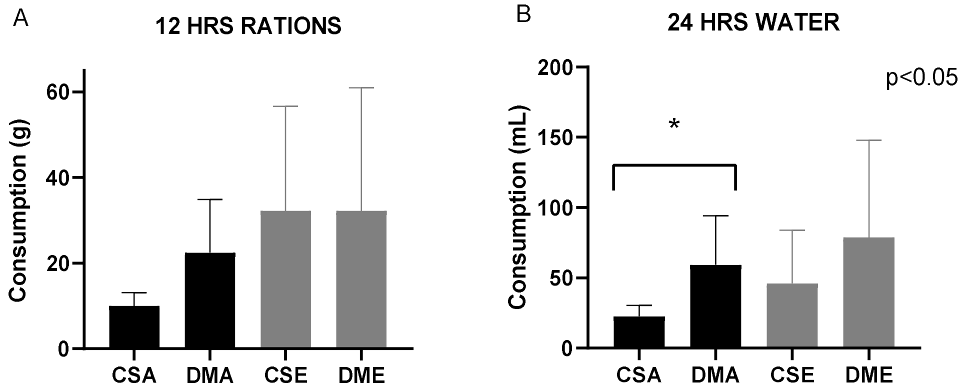

Fig 1.1: A Food consumption of the studied groups during 12 h in the metabolic cage. **B** Water consumption in the studied groups during 24 h in the metabolic cage. Values presented as mean ± DSVP. CSA n= 9, DMA n= 5, CSI n= 9 and DME n= 7. Kruskal-Wallis test. *P<0.05. 95% CI.

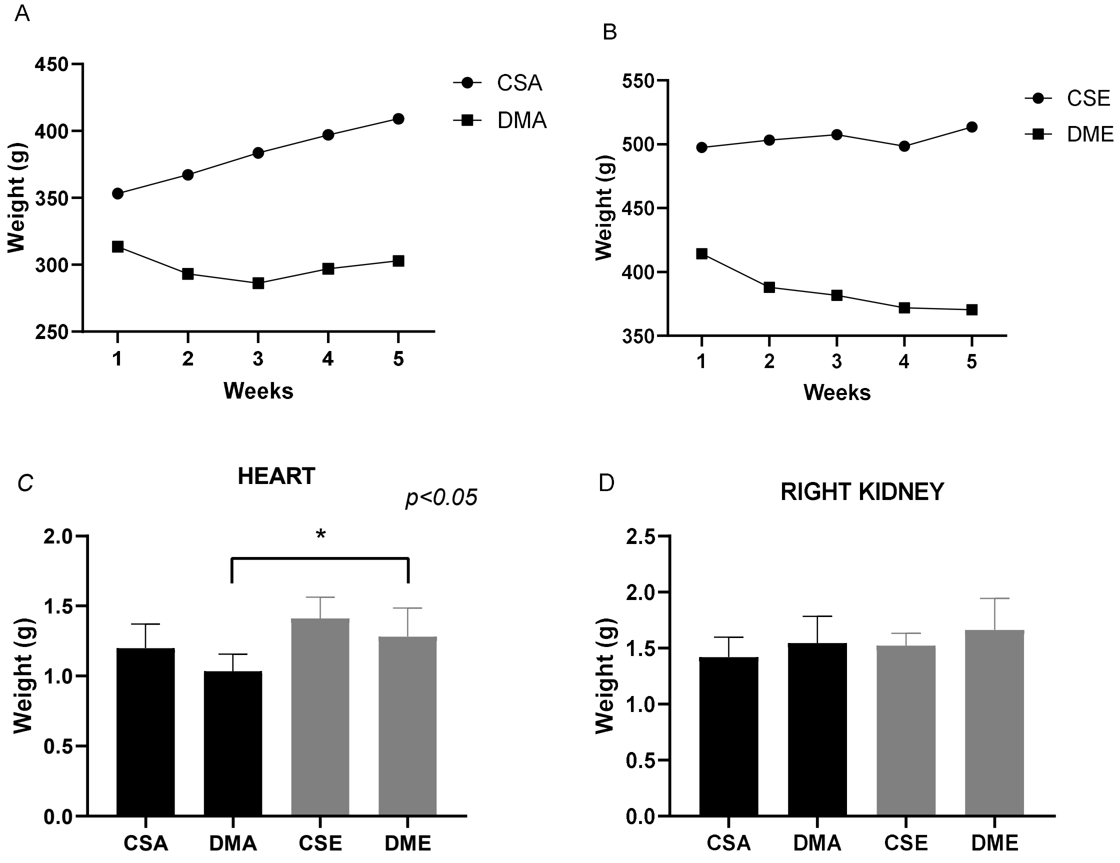

Fig. 1.2: A, B Graph of mean weight over 5 weeks of the DMA group (n= 5) versus CSA (n= 9) and DMI (n= 7) vs. CSI (n= 9), respectively. Values presented as mean. **C,**

D Graph representing the weight in grams of the heart and right kidney respectively, of the studied groups on the day of euthanasia. Values presented as mean ± DSVP. Kruskal-Wallis Test. *$P<0.05$. 95% CI.
