# Supplementary material for: Expression of TNFR1, VEGFA, CD147 and MCT1 as early biomarkers of diabetes complications and the impact of aging on this profile
Source: Sci Rep. 2023 Oct 20;13:17927. doi: 10.1038/s41598-023-41061-0 (PMC10589356; doi:10.1038/s41598-023-41061-0)
Supplement: Supplementary file 2 — Supplementary Information 2. [file 41598_2023_41061_MOESM2_ESM.pdf]

Bulbar, heart, kidney, blood and liver expression of target genes

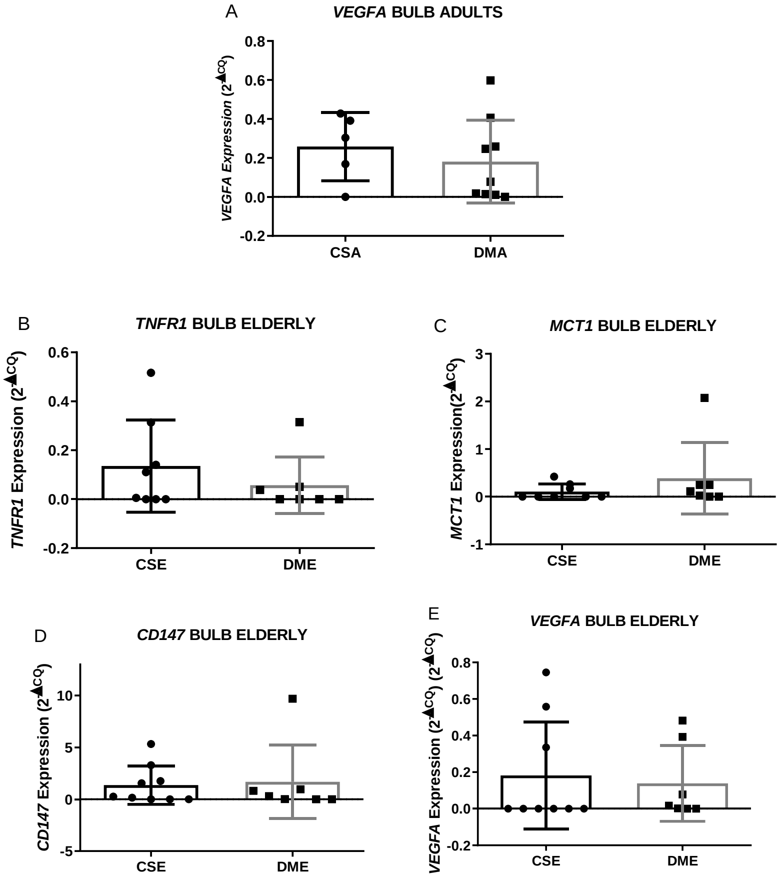

Fig. 2.1: Representative graphs of *VEGFA* expression in the brain bulb of the DMA group (**A**) and of *TNFR1*, *MCT1*, *CD147* and *VEGFA* in the DMI group (**B, C, D and E**). Data expressed as mean ± DSVP. Mann-Whitney Test. *p<0.05 vs. control. 95% CI.

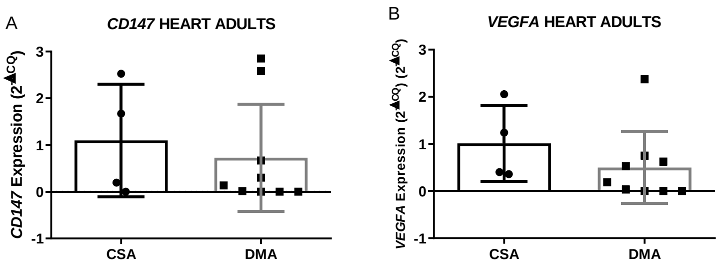

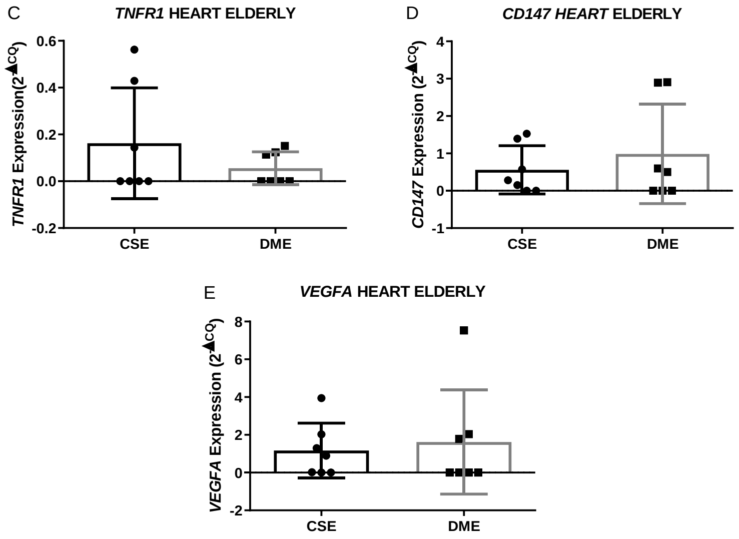

Fig. 2.2: Representative graphs of *CD147* and *VEGFA* expression in the heart of the DMA group (**A, B**) and *TNFR1*, *CD147* and *VEGFA* in the heart of the DMI group (**C, D and E**). Data expressed as mean ± DSVP. Mann-Whitney Test. *p<0.05 vs. control. 95% CI.

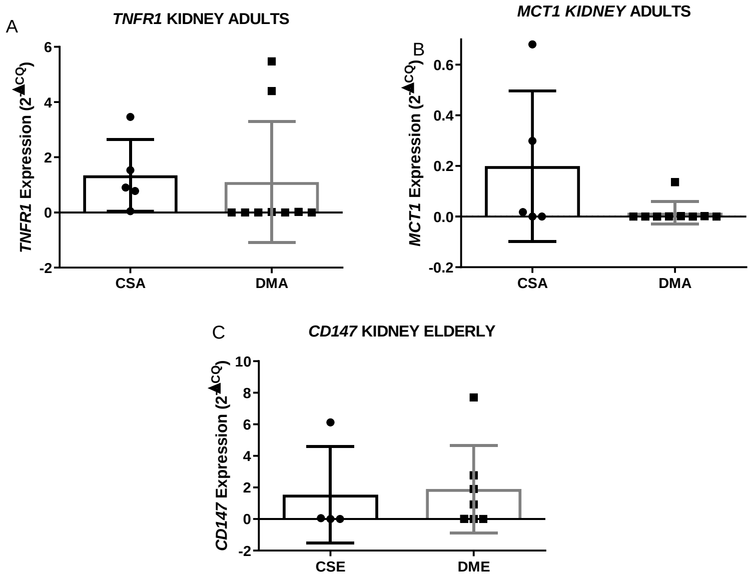

Fig. 2.3: *TNFR1* and *MCT1* expression in the DMA group (**A, B, C**) and of *CD147* in the DMI group (**C**). Data expressed as mean ± DSVP. Mann-Whitney Test. *p<0.05 vs. control. 95% CI.

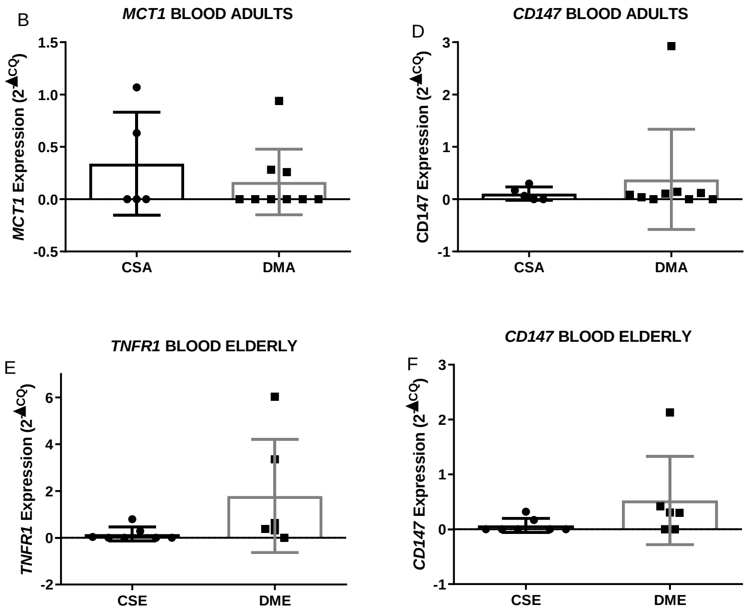

Fig.2.4: *MCT1* and *CD147* expression in the peripheral blood of the adult group (**A, B**) and of *TNFR1* and *CD147* in the elderly group (**C, D**). Data expressed as mean ± DSVP. Mann-Whitney Test. *p<0.05 vs. control. 95% CI.

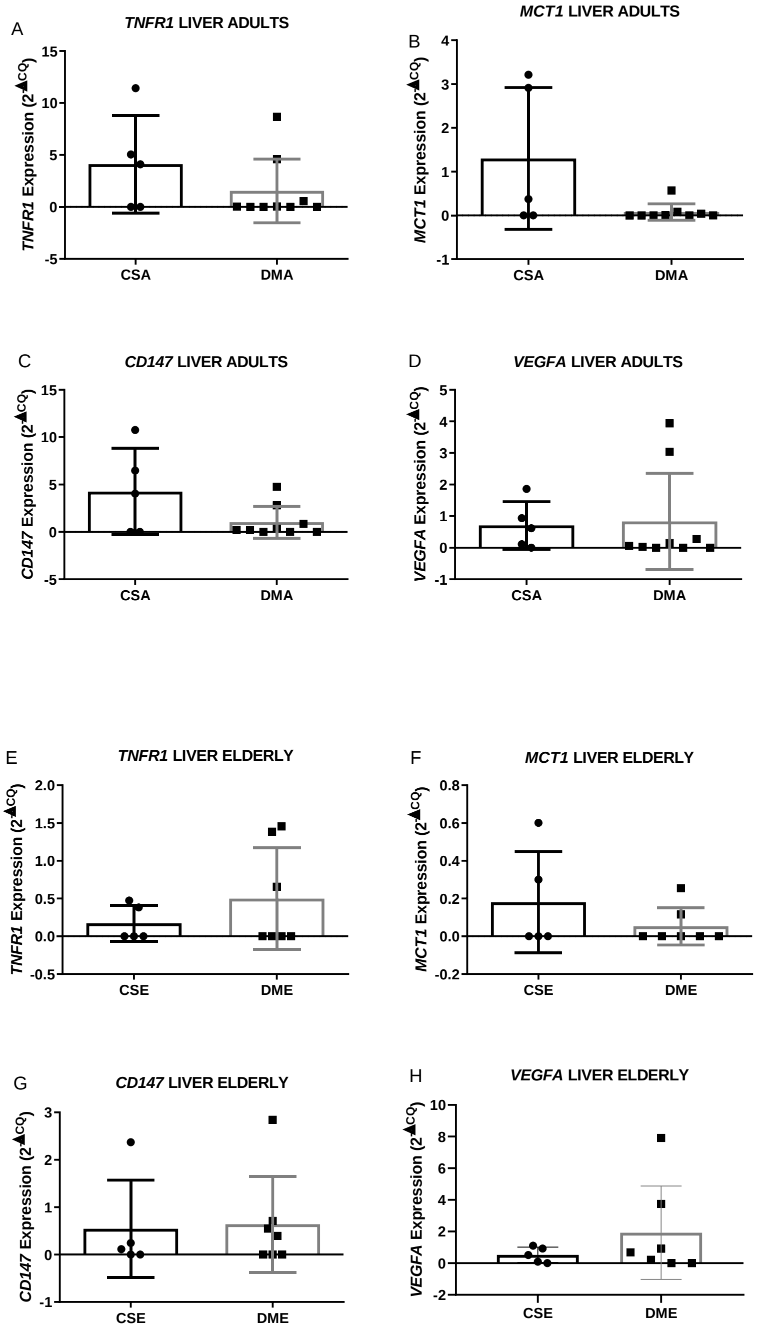

Fig 2.5: *TNFR1*, *MCT1*, *CD147* and *VEGFA* in liver tissue of the adult group (**A, B, C and D**) and of the elderly group (**E, F, G and H**). Data expressed as mean ± DSVP. Mann-Whitney Test. *p<0.05 vs. control. 95% CI.
