# Supplementary material for: Expression of TNFR1, VEGFA, CD147 and MCT1 as early biomarkers of diabetes complications and the impact of aging on this profile
Source: Sci Rep. 2023 Oct 20;13:17927. doi: 10.1038/s41598-023-41061-0 (PMC10589356; doi:10.1038/s41598-023-41061-0)
Supplement: Supplementary file 3 — Supplementary Information 3. [file 41598_2023_41061_MOESM3_ESM.pdf]

Gene expression profile in all the studied groups and tissues

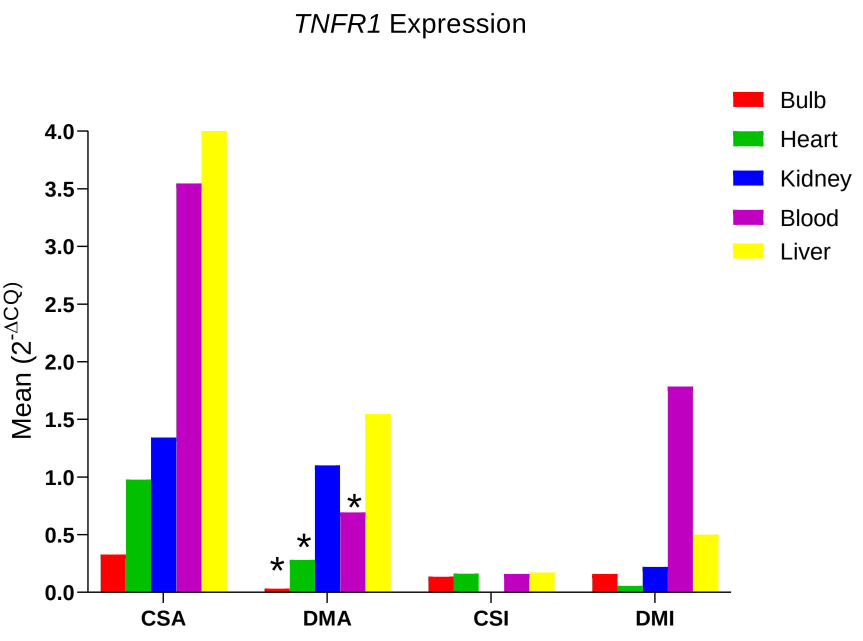

Fig.3.1: Representative graph of *TNFR1* expression in all the studied groups and tissues. Pooled values expressed as mean. Mann-Whitney Test. *p<0.05 vs. control. 95% CI.

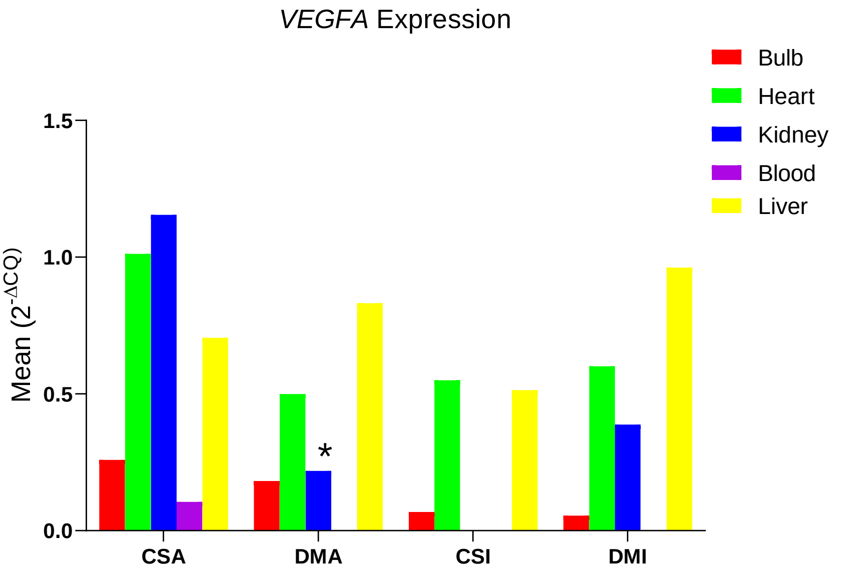

Fig.3.2: Representative graph of *VEGFA* expression in all the studied groups and tissues. Pooled values expressed as mean. Mann-Whitney Test. *p<0.05 vs. control. 95% CI.

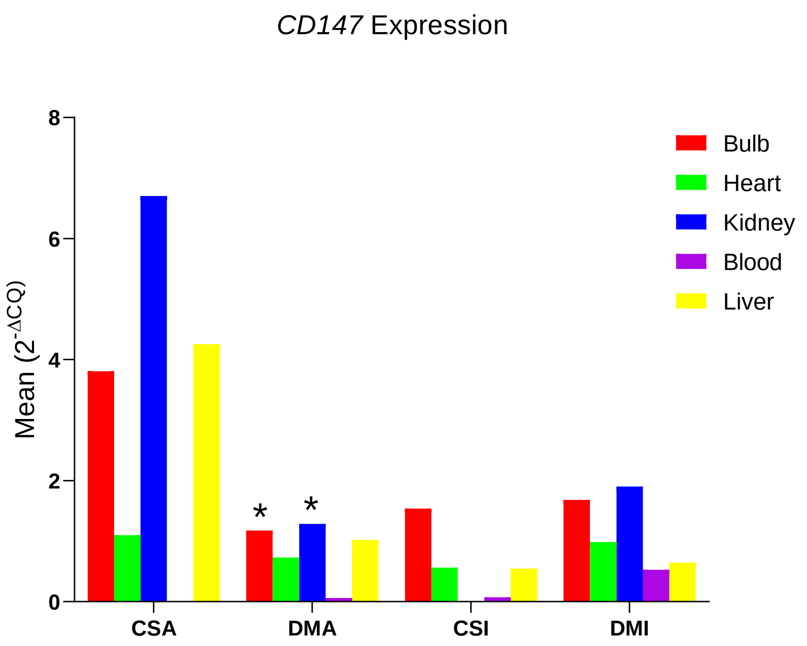

Fig.3.3: Representative graph of *CD147* expression in all the studied groups and tissues. Pooled values expressed as mean. Mann-Whitney Test. *p<0.05 vs. control. 95% CI.

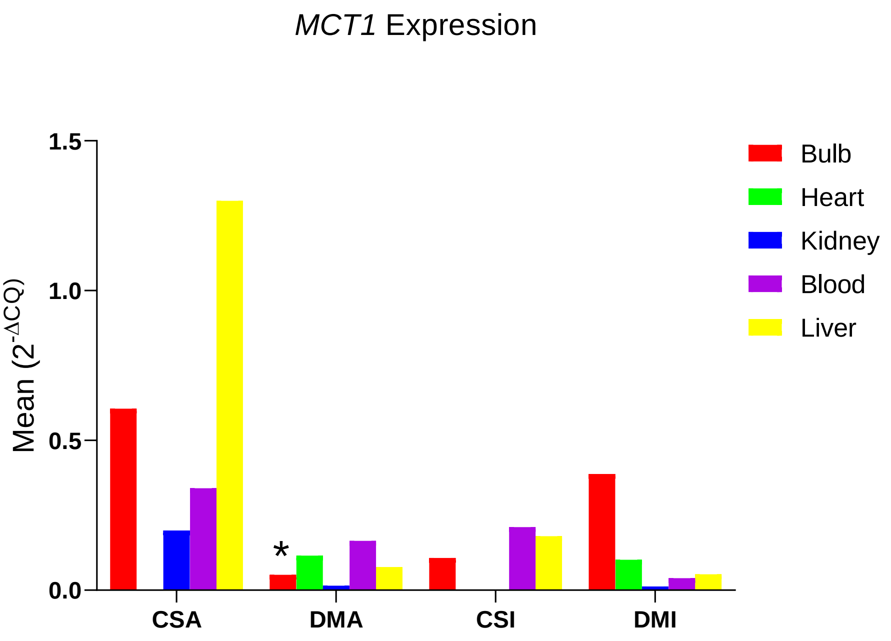

Fig.3.4: Representative graph of *MCT1* expression in all the studied groups and tissues. Pooled values expressed as mean. Mann-Whitney Test. *p<0.05 vs. control. 95% CI.
