# Supplementary material for: Expression of TNFR1, VEGFA, CD147 and MCT1 as early biomarkers of diabetes complications and the impact of aging on this profile
Source: Sci Rep. 2023 Oct 20;13:17927. doi: 10.1038/s41598-023-41061-0 (PMC10589356; doi:10.1038/s41598-023-41061-0)
Supplement: Supplementary file 4 — Supplementary Information 4. [file 41598_2023_41061_MOESM4_ESM.pdf]

Confirmation of amplicon size

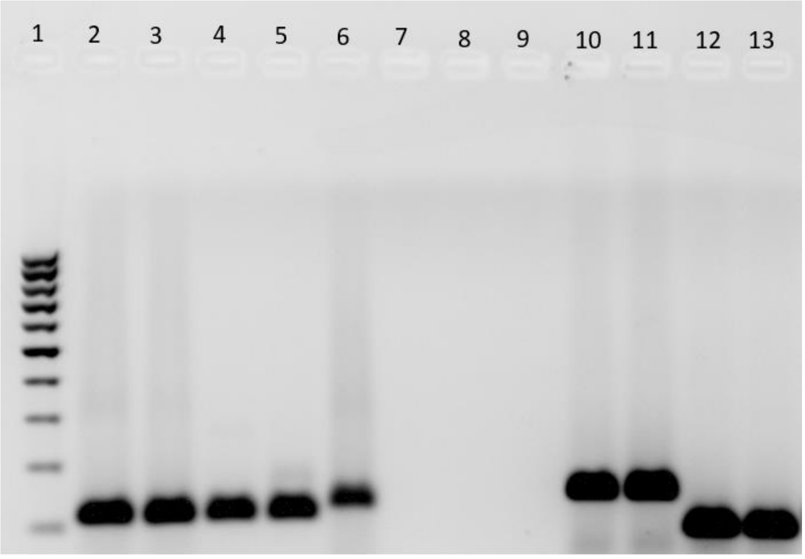

Fig 4 : Confirmation of amplicon size on agarose gel. *Ladder* 100 bp (BioLabs) (1), TNFR1 (2, 3, 4 and 5), VEGFA (6), PTX-3 (10 and 11) and FGF-23 (12 and 13). Lanes 7, 8 and 9: blank.
